# Prevalence of diabetes foot ulcers and associated factors among adult diabetic patients in three referral hospitals in Mogadishu, Somalia

**DOI:** 10.3389/fpubh.2023.1195483

**Published:** 2023-07-20

**Authors:** Abdulwahab M. Salad, Hodan A. Duale, Ismael M. Sheikh, Gallad Dahir Hassan, Abdiqani A. Farah, Abdi Gele

**Affiliations:** ^1^School of Public Health and Research, Somali National University, Mogadishu, Somalia; ^2^Somali Institute for Health Research (SIHR), Garoowe, Somalia; ^3^Norwegian Institute of Public Health, Garoowe, Somalia

**Keywords:** diabetes, ulcers, prevalence, hospital, Somalia

## Abstract

**Background:**

Diabetes mellitus (DM) causes significant morbidity and mortality in sub-Saharan Africa (SSA), including Somalia. Among diabetic patients, diabetic foot ulcers (DFUs) constitute the largest proportion of admissions, amputations, and mortality. The aim of this study is to assess the prevalence of diabetic foot ulcers and subsequently determine factors associated with it among diabetic patients at three major hospitals in Somalia.

**Methods:**

An institutional-based cross-sectional study was conducted among 193 diabetic patients between August and November 2022. All eligible diabetes patients who were attending De Martini Hospital, Madina General Hospital, and Deynile General Hospital during the study period were included in the study. Patients were interviewed using a structured questionnaire. We collected demographic, clinical, and behavioral variables from all participants. A bivariate and multivariable logistic regression model was fitted to identify factors associated with diabetic foot ulcer. An odds ratio with a 95% confidence interval was computed to determine the level of significance.

**Result:**

The mean age of the study’s participants was 50.9 ± 13.6 years. The prevalence of diabetic foot ulcer was 15%. Patients who were either overweight or obese (OR 4.63, CI: 2.08–10.30), had a lack of family support in managing diabetes (OR 3.33, CI: 1.74–6.36), and did not check their feet regularly were more likely to develop DFU (OR 1.99, CI:1.08–3.66).

**Conclusion:**

Increased body mass index, lack of family support, and not checking feet regularly were associated with DFUs. The high prevalence of DFUs and the plethora of needs of people with DFUs pose challenges for health care. A coordinated health care system is necessary to meet the needs of diabetic patients and prevent DFUs.

## Background

The rising prevalence of diabetes mellitus (DM) worldwide, with an estimated 642 million people with diabetes globally by 2040, is a global public health concern (1). In 2019, it was estimated that there were 19 million persons with diabetes in SSA, and the projected prevalence of DM to be 29 million in 2030 and 47 million by 2045 (2, 3). DM is associated with many systematic complications, including diabetic foot ulcers (DFU), which is an infection, ulceration, or destruction of the deep tissues of the foot. It is a devastating component of progression of diabetes with estimated 15% of patients with diabetes may develop DFUs, leading to more than 80,000 amputations per year in the United States (4). Diabetic foot ulcer (DFU) are defined as a “foot affected by ulceration that is associated with neuropathy and/or peripheral arterial disease of the lower limb in a patient with diabetes.” It is often caused by loss of glycemic control, peripheral neuropathy, peripheral vascular disease, and immunosuppression. A previous systematic review of the risk stratification systems for diabetic foot ulceration identified: previous lower extremity amputation, history of a foot ulcer, anatomic foot deformity, peripheral vascular disease, poor glycemic control and smoking (5). The lifetime risk of DFUs for patients with diabetes may reach up to 68 per 1,000 persons as reported by some studies (6). In Africa, the overall prevalence of foot ulcers was 13% and has increased over time, especially since 2001. Approximately 15% of patients with foot lesions underwent major amputation and 14.2% died during hospitalization. In patients with diabetic ulcers, neuropathy was the most common predisposing factor (7).

Somalia is a sub-Saharan African country that has experienced decades of armed conflict that have rendered the national public health care system dysfunctional. As a result, the health care system in the country is dominated by the private system, which is unaffordable for a large segment of the population (8–10). Although there is no representative data about diabetes in Somalia, the World Health Organization has estimated it at 5% in 2016, with 22% of the people in Somalia being overweight or obese (11). The 2021 data by the World Bank shows that the diabetes prevalence of the population between the ages of 20 and 79 in Somalia is 6.5% (12). The number of diabetes patients with foot ulcer is not yet known in Somalia due to the absence of both regular diabetes screening, and strict management for the known diabetes patients. The current study is the first of its kind investigating the prevalence of diabetes foot ulcers and associated risk factors among diabetes patients in Somalia.

## Methods

A hospital-based cross-sectional study was performed with 193 diabetic patients who attended the De Martini Hospital, Madina General Hospital, and Deynile General Hospital from August to November 2022. The first and second hospitals are referral public hospitals, while the third is a community hospital that provides free health services to a large catchment area in the northern part of Mogadishu. All are located in Mogadishu, the capital city of Somalia. The study population is composed of diabetic adult patients who come to the clinic for check-ups or are admitted to one of the three hospitals for diabetes related problems during the study period. The aim of this study was to assess the prevalence of diabetic foot ulcers and factors associated with them among diabetic patients in the three hospitals. All eligible diabetes patients who were attending the three hospitals during the study period were included in the study. Patients were interviewed using a structured questionnaire. DFUs were determined by reports of the history of a breach on the normal skin occurring as indurations, ulcerations, or changes of colors on the foot. Patients who were critically ill with a traumatic ulcer, patients admitted with diabetic ketoacidosis, patients with clinical suspicion of having Charcot foot, and diabetic patients who refused participation in the study were excluded. A validated, pretested, and structured questionnaire was used to gather information from the participants. The questionnaire was validated prior to application with three patients, and it was reviewed by two public health experts. The questionnaire included questions about sociodemographic characteristics, clinical variables, and behavioral factors.

The data were entered into Microsoft Excel version 16 and then exported into SPSS version 23.0 for analysis. Descriptive analyses were performed to summarize the characteristics of the study participants. To determine factors associated with diabetic foot ulcers, bivariable logistic regression was performed. Variables with a value of *p* < 0.25, in the bivariable analysis, were taken further to the multivariable logistic regression analysis. A *p*-value < 0.05 and a 95% confidence interval were considered statistically significant. Ethical clearance was obtained from the Institutional Review Board of the School of Public Health and Research, Somali National University. Written permission was obtained from hospital managers. Since most of the study participants could not write, verbal informed consent was obtained from them.

## Result

### Socio-demographic characteristics of respondents

A total of 193 adult diabetic patients were enrolled in the study. Of them, 76 (39.4%) were males, while 117 (60.6%) were females. The mean age of the study participants was 50.9 ± 13.6 years, and most of the study participants were in the age group 40–59 years. Regarding their educational status, 104 (53.9%) had no formal education, while 89 (46.1%) had formal education. More than half (50.2%) were unemployed, and the majority (75.6%) were urban residents. Of the total sample, 29 (15%, CI: 0.01–0.06) had diabetes foot ulcer during the study.

Of the total 193 study respondents, 69 (35.8%) had regular evaluation and treatment for diabetes mellitus. The majority, 170 (88.1%), had lived with diabetes for more than 10 years, and 54.4% had poorly controlled diabetes mellitus. Nearly half of the study respondents, 91 (46.2%), were either overweight or obese. Eighty-two (42.5%) participants had a family history of diabetes foot ulceration while 17.6% had Peripheral neuropathy (Table 1).

**Table 1:** Clinical factors of the study participants (*n* = 193).

Variable visit your physician regularly for evaluation and treatment of your diabetes	Frequency	Percent
Yes	69	35.8%
No	124	64.2%
*Duration of diabetes mellitus (year)* < 10 years	170	88.1%
≥ 10 years	23	11.9%
*Controlled diabetes*		
Yes	88	45.6%
No	105	54.4%
*Another known disease* Yes	89	46.1%
No	104	53.9%
*Type of known disease* Do not have known diseases	104	53.9%
Hypertension	49	25.4%
Others	40	20.7%
*Blood pressure*		
< 120/80	129	66.8%
120/80–139/89 and ≥ 140/90	64	26.9%
*Body mass index*		
18.5–24.9	102	52.8%
25–29.9 and ≥ 30	91	47.2%
*A family history of diabetes foot ulceration*		
Yes	82	42.5%
No	111	57.5%
*Foot ulceration currently* Yes	29	15%
No	164	85%
*Peripheral neuropathy* Yes	34	17.6%
No	159	82.4%
*Skin problems* Yes	39	20.2%
No	154	79.8%
*Nail problems* Yes	53	27.5%
No	140	72.5%
*Foot deformity* Yes	14	7.3%
No	179	92.7%

### Behavioral factors of respondents

The majority of participants (62.2%) did not receive family assistance during diabetes care, while nearly half (48.7%) reported that they always inspected their shoes for foreign objects. 30.1% of the respondents reported having walked outside barefoot.

Thirty-three (17.1%) respondents were smokers, while 138 (71.5%) of the participants stated that they engaged in physical exercise. Nearly half (46.6%) reported the type of exercise they engage in as walking, and more than half (50.3%) engage in physical activities fewer than four times per week. The behavioral factors of the respondents are shown in Table 2.

**Table 2:** Behavioral characteristics of study participants (*n* = 193).

Variable	Frequency	Percent
*Walk outside barefoot*		
Yes	58	30.1%
No	135	69.9%
*Inspect your shoes for foreign objects* Yes	94	48.7%
No	99	51.3%
*Inspect your feet daily* Yes	130	67.4%
No	63	32.6%
*Using moisturizer creams* Yes	131	67.9%
No	62	32.1%
*Moisturizing cream between your toes* Yes	116	60.1%
No	77	39.9%
*Family assistance during feet washing and moisturizing*		
Yes	73	37.8%
No	120	62.2%
*Regular physical activity* Yes	138	71.5%
No	55	28.5%
*Frequency of physical activity per week* Didn’t exercise	55	28.5%
< 4 times per week	97	50.3%
≥ 5 times per week	41	21.2%
*Type of exercise* Didn’t exercise	55	28.5%
Walking	90	46.6%
Others	48	24.9%
*Smoker*		
Yes	33	17.1%
No	160	82.9%
Using ill-fitting shoes		
Yes	31	16.1%
No	162	83.9%

Table 3 shows variables that are associated with diabetic foot ulcer after adjusting age, education, and employment. Overweight or obese people had almost five times higher odds of developing diabetic foot ulcer compared to diabetic patients who had a normal body mass index (OR 4.63, CI: 2.08–10.30). Similarly, people who do not receive family support in managing diabetes had over three times higher odds of developing DFUs (OR 3.33, CI: 1.74–6.36). Further, people who do not check their feet regularly were more likely to develop DFUs compared to those who did it (OR 1.99, CI:1.08–3.66).

**Table 3:** The variables associated with diabetic foot ulcer among diabetic patients (*n* = 193).

Variables	Diabetic foot ulcer		Model 1 OR(CI)	Model 2 OR (CI)	Model 3 AOR (95%I)
	Yes	No	COR (95% CI)	+Age only	Age, education, and occupation
Residence area					
Urban	18	128	1	1	1
	11	36	3.27 (1.66, 6.42)^*^	0.92 (0.39, 2.18)	–
Rural	16	66	4.12 (2.38, 7.12)^*^	1.51 (0.77, 2.97)	–
A family history of diabetes foot ulceration	13	98	1	1	1
Yes					
No					
Peripheral neuropathy					
Yes	20	14	0.70 (0.35, 1.38)	-	-
No	9	150	1	1	1
Body Mass Index					
18.5–24.9	21	81	1	1	1
25–29.9 and ≥ 30	8	83	10.37 (5.02, 21.43)^*^	5.36 (2.46, 11.67)^*^	4.63 (2.08, 10.30)
Blood pressure					
<120/80	11	118	1		
120/80–139/89 and ≥ 140/90	18	46	2.55 (1.48, 4.40)^*^	1	1
				0.70 (0.34, 1.46)	–
Duration of diabetes					
< 10 years	21	149	1	1	
≥10 years	8	15	1.87 (0.79, 4.42)	0.42 (0.13, 1.34)	
Get family assistance during diabetic care					
Yes	14	59	1	1	1
No	15	105	7.00 (4.07, 12.02)^*^	3.62 (1.95, 6.743)^*^	-
					1 3.33 (1.74, 6.36)^*^
Physical activity regularly					
Yes	16	122	1	1	1
	13	42	3.23 (1.73, 6.01)^*^	1.00 (0.45, 2.20)	–
No	7	26	3.71 (1.61, 8.55)^*^		
	22	138	1		
Smoker					
Yes				1.10 (0.42, 2.86)	–
No	12	46	3.83 (2.03, 7.23)^*^	1	1
Walk outside barefoot					
Yes				1.22 (0.58, 2.56)	-
No	17	118	1	1	1
Inspect your feet daily					
Yes	22	108	4.90 (3.10, 7.76) *	2.27 (1.27, 4.05)^*^	1.99 (1.08, 3.66)^*^
No	7	56	1	1	1

*Statistically significant.

## Discussion

The study shows that the prevalence of diabetes foot ulcers (DFUs) is 15% among study participants. A prior review of data from 19 African countries found that 13% of diabetes patients had foot ulcers (7). Despite the fact that the prevalence of DFUs in our sample is slightly higher than that observed in other parts of the world, Somalia is facing a formidable challenge in the management of DFUs, given that the country has one of the poorest health care systems in Africa. Therefore, like many other countries in Africa, the DFUs in Somalia may frequently progress to sepsis or gangrene, resulting in prolonged hospital stays and significant mortality (13, 14). A study shows that in the Africa continent, with poor resources, how to prevent and manage successfully DFUs is a major challenge (14). Large segments of the Somali people live in rural, while 26% are nomadic pastoralists that travel with their livestock often with barefoot. In this situation, it is not unexpected that the prevalence of DFUs in Somalia is 15%. Evidence shows that the diabetic foot complications resulting in amputation begin overwhelmingly with the formation of skin ulcers (15). Early detection and appropriate treatment of these ulcers may prevent up to 85% of amputation (15).

Factors that are associated with the DFUs in our sample include increased BMI, with people who are either overweight or obese having almost five times higher odds of developing DFUs. Literature documented a link between increased body mass index (BMI) and chronic DFUs (16–18). As high BMI drives number of functional impairments that are related to obesity and type 2 diabetes mellitus (19), the BMI > 25 may alter biological processes that are important in wound healing. For example, increased BMI is associated with impaired circulation which may compromise ulcer healing process (20), and impair oxygen delivery, thereby encouraging the growth of anaerobic microbes and fungi (21). Since Somalia does not have laboratory capacity to culture and conduct sensitivity tests these cases are often treated traditionally or not treated at all, thus eventually leading to amputation. A study in Tanzania, a country with much better health system than Somalia, shown that among patients admitted with DFUs, 33% had amputations, and 54% of those, with advanced pathology (Wagner Score ≥ 4), died within 1 year (22). Although there is no available data about overweight and obesity in Somalia, research on the Somali diaspora showed extremely high rates of obesity among Somalis, and a higher risk of DM (23). Combined with poor diabetes management, the DFUs may contribute to high rates of morbidity and mortality among people with DM in Somalia.

Variables such as long duration of diabetes, having high blood pressure, and walking barefoot have shown significantly higher odds of having DFUs in our sample, but this significance attenuated after we controlled the age, education, and occupation. However, people who do not receive family assistance for diabetes care have over three times the odds of having DFUs than those who do. A prior review of the literature stated that family support is an integral part of sustaining self-management behaviors and improving the health outcomes of diabetes patients (24). In Somalia, professional support services, such as nurse visits or disease education programs, are supposed to provide invaluable support for patients with diabetes are often unavailable. As a result, family members are recognized as a viable option in the care of DFU patients in conflict settings such as Somalia. There is empirical evidence about the association between insufficient social support from family members and poor diabetes self-management (25) and the fact that family support has a positive effect on patients’ self-management behaviors (25, 26). In Somalia, where professional support services are scarce, family shapes the environment in which self-care takes place and frequently plays an active role in managing the patient’s chronic illness. Family members are often the first to notice new symptoms, and most health problems are handled by patients and family members without consulting a health care professional (27). It is known that family support is linked with improved self-reported health and general well-being (28) as well as improved coping, quality of life, and glycemic outcomes (29). Our finding suggests that family support for diabetic patients, particularly those with DFUs, should be fostered in Somalia to prevent the adverse effects of diabetes, including DFUs.

Moreover, patients who do not inspect their feet daily had twice the odds of having DFUs as those who inspect their feet daily. According to the CDC, people who check their feet every day, can catch problems early and get them treated right away, thus reducing their risk of amputation (30). Adequate self-care can reduce the risk of lesions, infections, and amputation in people at risk of foot ulcer. The aforesaid self-care may include, but is not limited to, daily foot control, adequate hygiene, not walking barefoot, using appropriate footwear, cutting nails, early professional care for open foot wounds and lesions, and routine foot examinations by a trained professional to identify diabetic foot complications. However, these types of self-care require a high level of health literacy by the patients, which is a level of health literacy that Somali patients may not have (31). Further, there is no health education about self-care measures for foot ulcers among diabetes patients in Somalia. The lack of health literacy on foot care and/or clear daily foot care plans increases the risk of developing ulcers and amputations (32), while foot ulcers and amputations were found to increase in patients who did not adopt self-care measures (33). In this case, foot care knowledge and foot care practices are highly associated (34), which implies that when patients receive appropriate foot care guidelines and education, they will carry out the corresponding practices (35). Therefore, it is imperative for diabetes patients in Somalia to receive health education and self-care guidelines to prevent the adverse consequences of diabetic foot ulcers.

The important limitation of this study is its cross-sectional design, which makes it difficult to establish the causes. Moreover, most of the variables were self-reported, with a distinct possibility of both under- and/or over-reporting. However, this is the first study of its kind ever conducted in Somalia, thereby providing highly needed information for health providers and policymakers in Somalia to improve healthcare for diabetic patients and strengthen the national prevention strategy for diabetes foot ulcers. The study was performed with all diabetic patients who were attending the three major hospitals in the country from August to November 2022; thus, the study may be generalized to diabetic patients in Mogadishu.

## Conclusion

The prevalence of foot ulcers in Somalia is higher than that of many countries in the region. Lack of foot care among patients and the absence of family support were associated with the development of DFUs. Therefore, more intensive health education about footcare directed at diabetes patients and their families might improve the DFUs among diabetes patients in Somalia. Further, regular physical activity for diabetes patients may help them maintain a normal body weight, which may prevent DFUs and subsequent amputations.

## Data availability statement

The raw data supporting the conclusions of this article will be made available by the authors, without undue reservation.

## Ethics statement

The studies involving human participants were reviewed and approved by Somali National University School of Public Health and Research Institutional Review Board. Written informed consent for participation was not required for this study in accordance with the national legislation and the institutional requirements.

## Author contributions

All authors listed have made a substantial, direct, and intellectual contribution to the work and approved it for publication.

## Conflict of interest

The authors declare that the research was conducted in the absence of any commercial or financial relationships that could be construed as a potential conflict of interest.

## Publisher’s note

All claims expressed in this article are solely those of the authors and do not necessarily represent those of their affiliated organizations, or those of the publisher, the editors and the reviewers. Any product that may be evaluated in this article, or claim that may be made by its manufacturer, is not guaranteed or endorsed by the publisher.
